# Molecular Diagnostics and Detection of Oomycetes on Fiber Crops

**DOI:** 10.3390/plants9060769

**Published:** 2020-06-19

**Authors:** Tuhong Wang, Chunsheng Gao, Yi Cheng, Zhimin Li, Jia Chen, Litao Guo, Jianping Xu

**Affiliations:** 1 Institute of Bast Fiber Crops and Center of Southern Economic Crops, Chinese Academy of Agricultural Sciences, Changsha 410205, China; wangtuhong@caas.cn (T.W.); gaochunsheng@caas.cn (C.G.); chengyi@caas.cn (Y.C.); lizhimin@caas.cn (Z.L.); chenjia01@caas.cn (J.C.); guolitao@caas.cn (L.G.); 2 Department of Biology, McMaster University, Hamilton, ON L8S 4K1, Canada

**Keywords:** fiber crops, oomycete, molecular identification, mitochondrial DNA, PCR assay

## Abstract

Fiber crops are an important group of economic plants. Traditionally cultivated for fiber, fiber crops have also become sources of other materials such as food, animal feed, cosmetics and medicine. Asia and America are the two main production areas of fiber crops in the world. However, oomycete diseases have become an important factor limiting their yield and quality, causing devastating consequences for the production of fiber crops in many regions. To effectively control oomycete pathogens and reduce their negative impacts on these crops, it is very important to have fast and accurate detection systems, especially in the early stages of infection. With the rapid development of molecular biology, the diagnosis of plant pathogens has progressed from relying on traditional morphological features to the increasing use of molecular methods. The objective of this paper was to review the current status of research on molecular diagnosis of oomycete pathogens on fiber crops. Our search of PubMed identified nearly 30 species or subspecies of oomycetes on fiber crops, among which the top three species were *Phytophthora boehmeriae, Phytophthora nicotianae* and *Pythium ultimum*. The gene regions that have been used for molecular identifications of these pathogens include the internal transcribed spacer (ITS) regions of the nuclear ribosomal RNA gene cluster, and genes coding for translation elongation factor 1α (*EF-1α*) and mitochondrial cytochrome c oxidase subunits I and II (*Cox 1, Cox 2*), etc. We summarize the molecular assays that have been used to identify these pathogens and discuss potential areas of future development for fast, specific, and accurate diagnosis of oomycetes on fiber crops.

## 1. Introduction

Plant pathogens include diverse groups of organisms that can parasite and infect plants and cause diseases. These pathogens are among the main factors limiting crop yield and quality. Each year, it is estimated that between 20–40% of all crop losses globally are due to pre- and post-harvest plant diseases [1]. Plant pathogens are evolutionary very diverse, including various bacteria, viruses, fungi, and oomycetes. Together, it is estimated that globally, there are 1.5 million species of plant pathogens with most still undescribed. Among the 140,000 or so described fungal species so far [2], around 20% are plant pathogens, and together, fungal pathogens cause 70–80% of all plant diseases [3]. Given the right ecological conditions, most plant pathogens can reproduce rapidly and have the potential to cause large-scale disease outbreaks in a short time. At present, due to the limited knowledge on most pathogens when outbreaks occur, farmers typically apply broad-spectrum antimicrobial agents and/or disinfectants in an effort to control pathogen spread. However, such applications often result in low control efficiency, select drug resistant pathogens, and cause environmental problems that can also impact human health. Having a rapid and accurate detection and identification system of plant pathogens could allow for more targeted treatments and significantly improve treatment outcomes. Indeed, rapid and accurate detection of plant pathogens before or after crop planting can not only provide early warning for diseases and reduce the use of pesticides, but also help ensure the quality of agricultural products and improve environmental protection [4].

Traditionally, the diagnosis of plant pathogens relies on visible disease symptoms and microbial cultures. For example, for fungal and oomycete pathogens, the visible signs of pathogen propagules including spores, sclerotia, or mycelia present on the plant, or disease symptoms such as wilts, necrosis, chlorosis, cankers, scabs, blights, mildews, rusts, and rots caused by these pathogens are often used to identify disease agents [5]. With the rapid development of molecular biology, the diagnosis technology of plant pathogens has developed from the traditional morphological diagnosis to the current molecular diagnosis. The molecular detection techniques of pathogens include conventional polymerase chain reaction (PCR), nested PCR, real-time quantitative PCR (RT-qPCR), loop-mediated isothermal amplification assay (LAMP), colloidal gold detection, next-generation sequencing (NGS), and so on. Conventional PCR identifies pathogens by designing either specific primers that target certain group(s) of organisms or universal primers that amplify many organisms followed by sequencing. The advantages of conventional PCR include convenience, low cost, and high sensitivity. However, it can be time-consuming, and may be difficult to design primers to distinguish closely related pathogens. Nested PCR is an improvement of conventional PCR. It consists of two rounds of PCR amplification using two sets of primer pairs to improve the specificity and sensitivity. The general condition and cost of nested PCR are similar to those of conventional PCR. RT-qPCR is a method that adds fluorescence group into the PCR reaction system, uses fluorescence signal accumulation to monitor the whole PCR process in real time, and finally uses standard curve to quantitatively analyze the unknown template. This method is fast, very sensitive, and can provide quantitative pathogen data. However, it needs a specialized instrument, and its cost can also be high. Colloidal gold detection is a new type of immunolabeling technology, which uses colloidal gold as a tracer to detect antigens in pathogens and antibodies in infected hosts. This method has the advantages of being simple, rapid, and accurate, but the cost is high. LAMP is a method of nucleic acid amplification at a stable temperature. It requires designing four specific primers for six regions of the target gene and takes about 15–60 min to achieve 10^9–^10^10^ times nucleic acid amplification. The advantages of LAMP include being simple, having easy detection of products, and low cost, as well as being suitable for rapid detection of pathogens in both lab and field conditions. However, the requirements for primer designs are high and it is often difficult to design primers to separate closely related species. Thus, not all pathogens are suitable for LAMP detection. NGS has significant potential for diagnosis of all pathogens, including important eukaryotic plant pathogens. It can sequence hundreds of thousands to millions of DNA molecules at one time, and can detect both culturable and unculturable pathogens. However, the assembly and analysis of NGS data can be laborious and time-consuming, requiring specialized expertise [6]. 

At present, molecular diagnostics and detection of fungal and oomycete pathogens mainly rely on PCR-based technologies and use the following DNA markers: the internal transcribed spacer (*ITS*) regions of the nuclear ribosomal RNA gene (*rDNA*) cluster, β-tubulin, large ribosomal subunit (*LSU*) of the *rDNA*, translation elongation factor 1α (*EF-1α*), as well as the mitochondria-encoded cytochrome c oxidase subunits I and II (*Cox 1*, *Cox 2*) or NADH dehydrogenase subunit 1 (*nad1*) genes [7]. In general, for known and culturable pathogens, ITS primers are the most commonly used primers for detection of oomycete pathogens. However, for unknown and unculturable pathogens, NGS would be the method of choice [8].

For most of the 20th century, oomycetes were considered part of the Fungal Kingdom. Although the analyses of gene and genome sequences have revealed that oomycetes are evolutionary distinct from the true fungi, the scientific literature on fungi and oomycetes often overlap. This is especially the case in plant pathology. Consequently, oomycete and fungal plant pathogens often appear together in the same papers in broad surveys of plant diseases. For example, Cheng et al. [9] recently reviewed the current status of research on molecular diagnosis of fungal pathogens on bast fiber crops and included a few examples of oomycete pathogens in these crops. Indeed, there have been few systematic reviews on oomycete plant pathogens and no review on molecular detection of oomycete pathogens on fiber crops. The purpose of this review is to summarize the current state of our understanding on oomycete pathogens associated with all fiber crops and describe the molecular methods that have been used for their identifications. In the sections below, we first provide an overview of fiber crops (Section 2). This is then followed by a summary of oomycete pathogens (Section 3). In the fourth section, we describe the oomycete pathogens that have been reported so far from fiber crops. In the fifth section, we focus on the specific target DNAs and the molecular assays that have been used to identify specific oomycete pathogens on fiber crops. In the sixth section, we summarize the current molecular methods reported for detecting oomycete pathogens in other crops. We finish by providing a brief summary of the progresses and discuss potential future areas of research and development.

## 2. Fiber Crops

Fiber crops are an important group of economic crops. They produce fiber as raw materials for a diversity of applications. To be economically viable, fiber crops must be able to produce large quantities of cellulose that can be relatively easily extracted for downstream processing. About 2000 species of plants from around the world have been reported as sources of natural fibers for commercial applications. However, only a small number of these plant species are commercially cultivated, and these cultivated species produce nearly 90% of the world’s natural fiber [10]. Based on FAO data, Asia and America are the main production areas of fiber crops in the world, with the Americas producing about 50% of the global total fiber, and Asia producing about 33% of the global output.

According to the part of the plant from which fibers are extracted, fiber crops can be divided into the following types: seed fiber (cotton, coconut husk coir, kapok, milkweed, luffa), bast fiber (flax, hemp, kenaf, jute, nettle, and ramie), leaf fiber (sisal, abaca, yucca, phormium, bowstring hemp and henequen), grass fiber (silvergrass, reed, and bamboo), palm fiber (windmill palm, Palmyra palm), and woody fiber (jarrah) [11]. However, it should be noted that aside from being sources of natural fiber, most fiber crops also contain other parts that have been used for a diversity of purposes, including food and food additives for humans, animal feed, raw materials for biofuel production, and fuel for heating [12]. 

In addition to the above-mentioned roles of fiber crops to humans and human welfare, the medicinal properties of several traditional fiber crops are also attracting increasing attention. For example, hemp (*Cannabis sativa*) of the bast fiber category contains a diversity of pharmacologically active compounds, some of which have been used to treat chronic rheumatic arthritis, glaucoma, asthma and mental disorders in humans. Flax has medicinal values as a skin moisturizer, pain relief, and in treatments of lung disease and diuresis [13]. Similarly, ramie, another bast fiber crop, can stanch bleeding, relieve pain, reduce inflammation, and slow cancer cell proliferations [14]. In addition, hemp and ramie fibers have been developed into environment-friendly mulch for crop and vegetable fields. Such mulch has several desirably properties, including being biodegradable, having strong permeability and high-water retention ability, and promoting the growth of beneficial soil microorganisms. Indeed, such natural-fiber based mulch has been considered an ideal substitute for petroleum-based polyethylene plastic covers [15]. Table 1 summarizes the main fiber crops, including their geographic distributions, habitats, commercial use, and main oomycete diseases.

Among the cultivated fiber crops, cotton (*Gossypium* spp.) is the most dominant in terms of world fiber production. The current estimate for world production of cotton is about 25 million tons [16]. Among the four types of cotton, *Gossypium hirsutum,* commonly known as upland cotton, Mexican cotton and Bourbon cotton, accounts for 90% of the world’s total cotton production. India, USA, and China are the world’s top three producers of cotton, with USA being the largest cotton exporter. Hemp fiber is stronger and more durable than almost any other natural fiber. However, hemp is a minor global crop in term of fiber production, with approximately 91,055 hectares (ha) planted in 2016. However, the value of hemp market was estimated to be very high, at $3.9 billion in 2017 and the hemp seed segment is predicted to grow at a compound annual growth rate of 17.1% through at least 2025 [17]. Similarly, the strength of flax fiber of the bast fiber category is twice that of cotton fiber and five times that of wool fiber. Under wet conditions, the strength of flax fiber is increased by 20% [18]. Based on statistics from the Food and Agriculture Organization of the United Nations (http://www.fao.org/faostat/, FAOSTAT), in 2018, world production of flax (linseed) was 3.18 million tons, led by Kazakhstan with 29% of the global total. Ramie is another important fiber crop of the bast fiber category. Ramie is one of the oldest fiber crops, having been used for at least six thousand years, and is primarily used for fabric production. More than 90% of the world’s ramie planting area is in China [19]. In addition, sisal is also an important member of fiber crops. Global production of sisal fiber in 2018 amounted to 198 thousand tons of which Brazil, the largest producing country, produced 80,042 tons based on FAOSTAT.

## 3. Oomycetes

Oomycetes belong to the kingdom Stramenopila [20,21]. Oomycetes share a range of morphological features with fungi, but they possess various unique characteristics which set them apart from true fungi. Specifically, in addition to their significant DNA sequence divergence, oomycetes differ from fungi in their cell structural, genetic, physiological and biochemical characteristics. For example, oomycetes mainly exist as diploids; their cell walls are primarily composed of cellulose and β-1,3-glucan, not chitin; their mitochondria possess tubular cristae; and their hyphae are always nonseptate [22].

**Figure 1:**
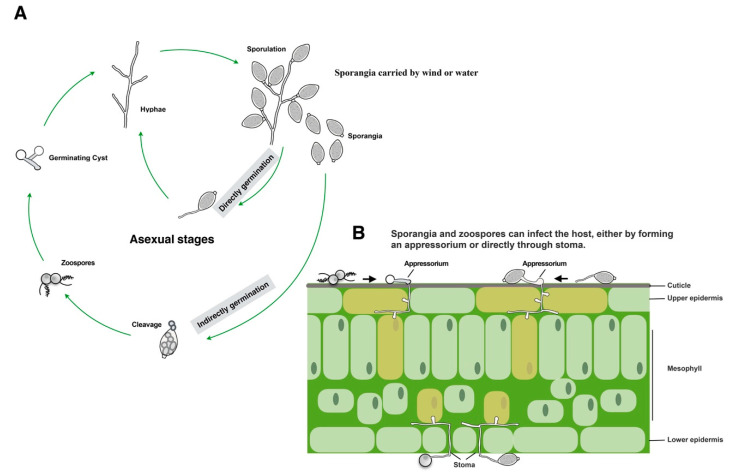
Diagram depicting the life cycle and pathogenesis of *Phytophthora* species (**A**) Typical asexual life cycle of *Phytophthora*. (**B**) Leaf colonization and invasion pattern.

Oomycetes can be found in diverse ecological niches including in marine, freshwater, and terrestrial environments [23,24]. Oomycete plant pathogens can exhibit biotrophic (e.g., *Hyaloperonospora arabidopsidis*), necrotrophic (e.g., *Pythium ultimum*), or hemibiotrophic (a combination of both, e.g., *Phytophthora palmivora*) lifestyles. Consequently, the pathogenesis of different oomycete pathogens may differ [25]. Among the oomycete pathogens, those in the genus *Phytophthora* are among the best studied, including their life cycles. Figure 1 shows the typical life cycle and pathogenesis of *Phytophthora* species. *Phytophthora* species reproduce asexually by producing sporangia that can be dispersed by wind and water. In response to cold shock conditions, sporangia cleave into zoospores, which swim, encyst, and germinate to form mycelia or a specialized infection structure (appressorium) on plants or on hydrophobic surfaces [26,27]. Sporangia can also germinate directly to produce mycelia or form an appressorium [28]. Both sporangia and zoospores are important for dissemination and host infection (Figure 1) [29]. 

Many oomycetes are important plant pathogens, causing severe diseases and crop losses. They can attack seeds, seedlings, and/or adult plants, and infect roots, leaves, shoots, stems, woody tissues, fruits, and/or flowers. They can enter host plants through direct penetration, through natural openings such as stomata, or through wounds. Among the diverse oomycete pathogens, those in the genera *Phytophthora* and *Pythium* are the most destructive plant pathogens known. They can infect different tissues and organs of plants, resulting in rot, wilt, and eventual collapse of whole infected plants. There are up to 90 species in the genus *Phytophthora* and 120 species in the genus *Pythium.* These oomycete pathogens have broad host ranges [26,30]. Examples of oomycete pathogens causing severe crop losses and human hardships include the potato late blight caused by *Phytophthora infestans* in the 1840s that resulted in Irish famine, and jarrah dieback in Australia caused by *Phytophthora cinnamomi* that infected over four hundred plant species belonging to forty different families, with the most severely affected belonging to the families Proteaceae, Leguminoseae, Epacridaceae, Myrtaceae and Xanthorrhoeaceae. [31,32]. In contrast, although *Pythium* spp. can cause pre-emergence damping off, resulting in reductions in plant growth and crop yield, *Pythium* pathogens generally are not lethal to mature plants. However, in recent years, researchers have found that certain *Pythium* pathogens can also cause lethal diseases to plants, such as root rot in the bast fiber crop ramie, caused by *Pythium vexans* (=*Phytopythium vexans*) [33].

## 4. Oomycete Pathogens of Fiber Crops

As shown in Table 1, most fiber crops can grow in a range of geographic regions and ecological niches. Each of these fiber crops is susceptible to a variety of oomycete pathogens. Table 2 summarizes the oomycete pathogens found so far on fiber crops and the types of diseases that these oomycete pathogens cause. 

As shown in Table 2, two genera (*Phytophthora* and *Pythium*) of oomycetes contained some of the most dominant plant pathogens, impacting plants and agricultural crops all over the world. The main pathogenic species in these two genera include *Phytophthora boehmeriae*, *Phytophthora cinnamomi*, *Phytophthora capsici*, *Phytophthora nicotianae*, *Phytophthora palmivora*, *Pythium aphanidermatum*, *Pythium ultimum*, and *Pythium vexans*.

For fiber crops, *Phytophthora boehmeriae* causes a variety of diseases such as leaf blight and root rot of cotton, as well as leaf blight of ramie and paper mulberry. In addition, it can cause gummosis and canker, brown rot (fruit) and root rot diseases in non-fiber crops and tress such as black wattle, citrus, black button, Mexican yellow pine, etc. [32]. *Phytophthora cinnamomi* causes a root rot or dieback and is one of the world’s most invasive pathogens, so far reported in more than 70 countries around the world. It can infect about 5000 species of plants, including 4000 Australian native species, including important agricultural and forestry plants, such as avocado, chestnut, macadamia, oak, peach, pineapple, and the fiber crop jarrah. The main site of infections are fine and fibrous roots causing root rot, as well as stems, causing stem cankers [57,58]. *Phytophthora capsici* is an important plant pathogen that causes blight and fruit rot of peppers and other important commercial crops, including cantaloupe, cucumber, watermelon, bell pepper, tomato, snap beans, lima beans, and the fiber crop sponge gourd [37]. In severe cases, the disease can cause 100% crop loss [59]. *Phytophthora palmivora* infects multiple hosts, including those of economic significance such as cacao, coconut, papaya, mango, and black pepper fruit rot or koleroga, making this a pathogen of great concern. In Italy, China, Sri Lanka, Mauritius, Sumatra, and India, *P. palmivora* causes bud-rot of palms and sisal zebra spot disease of fiber crops [34,42]. It has been estimated that in typical years, 10–20% of all cacao is lost due to Phytophthora Pod Rots (PPR) caused by *P. palmivora*, but with as high as 75% losses in some regions [60]. *Phytophthora nicotianae*, has a broad host range comprising 255 genera from 90 families of plants, including tobacco, onion, tomato, ornamentals, pepper, citrus plants, and fiber crops such as cotton, windmill palm, and sisal. This pathogen can cause root rot, crown rot, fruit rot, leaf infection, and stem infection [34,42,57].

Among the *Pythium* species, *Pythium aphanidermatum* is a soil borne plant pathogen, has a wide host range, including soybeans, beets, peppers, chrysanthemum, cucurbits, and fiber crops such as okra and hemp. It causes damping off, root rot, and crown wilt [43,44,45]. *Pythium ultimum*, which causes damping off and root rot more than 300 diverse hosts, including corn, soybean, strawberry, wheat, Douglas fir and ornamentals, has caused huge economic losses to the country [61]. It can cause flax damping off, hemp crown and root rot and cotton damping-off of fiber crops [53,54,55]. *Pythium vexans* (=*Phytopythium vexans*) is a causative agent of patch canker, damping-off, and crown, stem, and root rot, in more than 50 economically important plants including various vegetables, fruit trees, flowers, tobacco, tea, sugarcane, cucumber, sweet potato, wheat, corn, strawberry, and fiber crop such as ramie. In recent years, *P. vexans* was found to cause brown root rot of ramie, resulting in >40% yield loss in some ramie plantations [33].

As shown in Table 2 and described above, oomycete diseases have become an important factor limiting the yield and quality of fiber crops, causing devastating consequences for the production of these crops in many regions. Thus, it is very important to be able to detect the pathogens quickly and accurately in the early stages of infection. Such information would allow farmers and agronomists to develop effective control measures as early as possible to reduce crop losses by these diseases.

Conventional identifications of oomycete pathogens involve isolating oomycete pathogens from infected plants and examining their morphological characteristics, such as sporangium, oogonium, antheridium and oospores. In general, the isolation and identification of oomycetes on fiber crops are similar to those on other crops, mainly rely on culture-based identification system that involves isolating and culturing the pathogens from the diseased tissues and soil, followed by morphological and/or molecular characterizations. Specifically, the diseased plant tissues are typically washed with tap water, dried with absorbent paper, surface-disinfected with ethanol or another disinfectant, and the tissue at the junction of diseased and healthy parts removed for culturing on select artificial media. A common selective medium for isolating oomycetes from the diseased tissue or soil is PARP [62]. To isolate oomycetes from the soil, there are two commonly used methods: the bait method and the dilution plating method [63]. After oomycete growth on media, hyphal tip cultures or zoospores are harvested and purified. They are then transferred to different culture conditions to induce the productions of oospores, sporangia and zoospores for morphological identifications. At the genus level, morphological features are often sufficient to distinguish oomycete genera. For example, the spores of *Phytophthora* are mainly ovate or pear shaped, with papillae, shed or not, while the sporangium of *Pythium* is mainly spherical, without mastoid and falling off. In *Phytophthora* the zoospores differentiate in the sporangium, whereas in *Pythium* the zoospores form in a vesicle that bulges out from the opening of the sporangia. Some species of *Phytophthora* such as *P. infestans*, *Phytophthora cactorum*, and *P. palmivora* can be distinguished from each other based on their microscopic morphology. However, *Pythium* species are frequently difficult to identify to the species level using morphological characteristics alone.

Using traditional methods, He et al. found that the main pathogen causing ramie blight diseases was *P. cactorum* [35,36]. Similarly, Brown and Mercer found that the main pathogen causing flax root rot disease was *Pythium intermedium* [49]. However, detection and identification of oomycete pathogens using traditional approaches require abundant knowledge and experience working with the group of organisms and those methods often take a long time to complete. In addition, closely related species have similar morphological and reproductive features, making it difficult to separate them. Furthermore, those methods often require equipment such as specialized media and incubators that are not available in field conditions. For example, in wet weather conditions, *Phytophthora boehmeriae* is usually the primary pathogen causing cotton and ramie blight. However, other microorganisms such as those in oomycete genus *Pythium*, and in fungal genera *Fusarium* and *Rhizoctonia* are found on infected tissues. As *P. boehmeriae* grows much slower than other pathogens, it can be difficult to isolate and identify the primary pathogen for the diseases [31]. With the rapid development of molecular biology, molecular approaches have led to greater confidence and accuracy in the identification of plant pathogenic oomycetes. The most prevalent molecular method relies upon polymerase chain reaction (PCR), which has long been used in the field of plant pathology, including for identifying the pathogens of fiber crops [64]. 

Using various molecular detection techniques, more and more oomycetes have been identified as causal agents of diseases on fiber crops. These studies have shown that one pathogen can infect multiple fiber crops (Table 2). In addition, a diversity of pathogens can infect the same fiber crop and cause similar disease symptoms. For example, based on molecular testing, cotton blight could be caused by several oomycete pathogens in the genus *Phytophthora*, including *P. boehmeriae*, *P. palmivora*, *P. drechsleri*, *P. cactorum,* and *P. nicotianae.* Among these pathogens, *P. boehmeriae* was the most prevalent agent for cotton blight [31]. Interestingly, *P. boehmeriae* can also cause ramie blight [31,32]. Similarly, *Phytophthora palmivora* could cause windmill palm bud and root rot and is a pathogen of cotton causing cotton blight [31,42].

## 5. Target DNA Selection, Molecular Assays and Phylogeny of Oomycete Pathogens on Fiber Crop

A number of gene fragments have been used for molecular identification of oomycete pathogens of fiber crops (Table 2). They include gene fragments from both the nuclear and mitochondrial genomes. The commonly used ones such as *ITS*, *LSU rRNA*, *SSU rRNA*, and *EF-1α*, are similar to those used for detecting fungal pathogens (Table 2, ref. [9]). These markers were often chosen mainly because sequence variations in these DNA fragments were effective for distinguishing closely related species. In addition, these gene fragments have been commonly used for phylogenetic and taxonomic studies of oomycetes. Below we briefly summarize the main DNA fragments and the specific molecular techniques that have been used to identify oomycete pathogens impacting fiber crops.

### 5.1. ITS—Conventional PCR

Most of the diagnostic assays designed to detect and identify oomycete pathogens were developed based sequence variation at specific fragments of the *rDNA* cluster. Similar to that in fungi, the *rDNA* gene cluster in oomycetes includes three highly conserved ribosomal RNA subunits—encoding genes, namely the *5.8S rRNA*, *18S rRNA* (also called the small subunit RNA or *SSU RNA*) and the *28S rRNA* (i.e., the large subunit RNA or *LSU RNA*). The segments between *18S* and *5.8S*, and between *5.8S* and *28S* are called ITS regions, including *ITS1* and *ITS2* [65]. *rDNA* evolves relatively slowly and has a wide range of conserved and variable regions, which provides convenience for the design of broad range and species-specific primers for oomycete molecular detection. Using the sequence variability feature within the *rDNA* gene cluster, Matsumoto et al. [66] used ITS1 and ITS4 primers originally designed by White et al. [67] to amplify *ITS* regions, including the 5.8S gene of *Pythium* species, followed by sequencing and identification based on ITS sequences. As shown in Table 2 and Table 3, more than half of the molecular detection studies used the ITS region and the universal ITS primers (e.g., ITS1 and ITS4) for the detection of oomycetes infecting fiber crops.

However, for efficient detection, it is preferable that the detection of a specific pathogen can be accomplished through a one-step process, without involving DNA sequencing and sequence comparisons. For this purpose, the ability to design specific-specific primers is the key. This is especially needed when multiple closely related pathogens can cause the same disease in a crop. Due to the presence of both highly conserved and variable regions within and around the ITS regions, these regions have served as excellent regions for developing primers to suit different needs, from universal to genus and species-specific primers. For example, five species of *Phytophthora* have been reported to infect cotton (*P. boehmeriae*, *P. palmivora*, *P. drechsleri*, *P. cactorum* and *P. nicotianae*), and they can be amplified by using conventional PCR with primers DC6 and ITS4, but only *P. boehmeriae* isolates yielded amplification products with primers PB1 and PB2 developed for the ITS regions [31].

### 5.2. Non-ITS Nuclear Genes—Conventional PCR

While the ITS regions of the rDNA have been the most commonly used for identification of oomycetes, for certain closely related species in both *Phytophthora* and *Pythium*, the ITS regions may not be appropriate for developing species-specific molecular markers. Such a problem could be due to a low level of sequence divergence between closely related species and/or a high level of intraspecific sequence variation. Under such circumstances, alternative markers have been developed, include the nuclear-encoded housekeeping genes *EF-1α* or *SSU rRNA* and *LSU rRNA* genes of the *rDNA* (Table 2 and Table 3) [7].

The *EF-1α* gene is a conserved single-copy nuclear protein-coding gene with low intraspecific variations in DNA sequences [68]. It is a secondary DNA barcode for many groups of fungi and fungus-like organisms, often used in phylogenetic studies of divergent fungal and oomycete groups. Although the database of *EF-1α* sequences is not as large as that for ITS sequences, *EF-1α* often contains more variable nucleotide sites than that of ITS and thus can be particularly useful for separating closely related organisms [69,70]. For example, Maizatul-Suriza et al. analyzed 43 *EF-1α* sequences of *P. palmivora* and other *Phytophthora* species from different hosts. They demonstrated findings similar to that based on ITS sequences, with low intraspecific variations in DNA sequences, but a high level of phylogenetic variation across species in the *Phytophthora* genus [68]. Similar to *EF-1α*, *SSU* and *LSU* also have obvious variability among species [7,71]. However, the amount of variation was lower than that of the ITS locus and *EF-1α* gene fragment. For example, Schroeder et al. indicated that phylogenetic analyses with *SSU* and *LSU* with broad sampling of *Pythium* revealed relatively limited support for many clades within the genus [7].

### 5.3. Mitochondrial Genes—Conventional PCR

In 2003, the international DNA barcoding initiative started, with the objective of identifying a universal barcode for all species on Earth. The first proposed barcode was the mitochondrial *Cox 1* (syn. *COI*) gene, which was broadly accepted for taxa identification in the animal kingdom [72]. Compared with nuclear genes, the mitochondrial gene is typically uniparentally inherited, lacks heterozygosity, and is relatively straightforward to analyze. In addition, in certain groups of eukaryotes, the mitochondrial genome has a faster evolution rate than that of the nuclear genomes, thus it can provide a greater degree of variation among species. Its high copy number in cells also makes *mtDNA* an attractive target for developing highly sensitive markers for analyses. Most fungal lineages harbor mitochondria, and in general, fungal mitogenomes usually contain the following protein-encoded genes: *atp6*, *atp8*, *atp9* (encoding subunits of ATP synthase), *cob* (encoding cytochrome b), *cox1-3* (encoding cytochrome oxidase subunits), *nad1-6*, and *nad4L* (encoding the NADH dehydrogenase subunits) [73,74]. 

There have been several studies of using mitochondrial DNA for detection and quantification of oomycetes. Tooley et al. developed the first mitochondria-based assays for plant pathogenic oomycetes in 2006. They designed primers and probes to detect *P. ramorum*, which infects a large number of trees [75]. Currently, about 20 oomycete mitogenomes are available in the GenBank database with a significant portion representing plant pathogenic species. The barcoding potential of *Cox 1* gene for oomycetes was confirmed by Robideau et al. in 2011 [76], and it showed that in some cases *Cox 1* was more variable and had a higher discrimination power than ITS.

Among the oomycete pathogens infecting fiber crops, *P. boehmeriae*, *P. capsici*, *P. elongate,* and *P. myriotylum*, *P. phragmatis*, and *P. sylvaticum* have been investigated using mitochondrial marker genes *Cox 1* and *Cox 2* (Table 2). In addition, several genetic markers are available for *P. boehmeriae* including *Cox 2*, *Nad 9*, *Rps 10*, and *Sec Y*. Similarly, signature sequences for *P. capsici* was found at *Cox 1*, *Cox 2*, *Nad 1* and *Nad 5* genes [32,37]. Indeed, Kulik et al. recently introduced detailed mitochondrial gene markers for detecting various plant pathogenic fungi and oomycetes, such as *Cox 2* for *Fusarium culmorum*, *Cox 1* and *Cox 2* for *P. ramorum*, *Cob* for *Fusarium graminearum* s.s., *atp9* for *Phytophthora* species [77].

### 5.4. mtDNA-RT-qPCR Technology

In 1996, Applied Biosystems in the USA first introduced RT-qPCR technology. Three years later, the technology was used for the first time in plant pathology research [78]. In 2002, Schaad et al. [79] introduced the technology to detect plant pathogenic fungi. In recent years, RT-qPCR technology has been widely used for the identification and detection of plant pathogens all over the world. Compared with conventional PCR, RT-qPCR is faster, more sensitive, more specific, and can distinguish the subtle differences among closely related pathogens. There are two main detection methods used in RT-qPCR, the SYBR Green method and the TaqMan method. The TaqMan method requires a specific probe that’s unique to the target organism. Overall, the SYBR green dye method is more widely used than the TaqMan method. The RT-qPCR technology has been used for detecting several oomycete pathogens in fiber crops (Table 2 and Table 3). For example, in 2019, Kunadiya et al. [38] used SYBR Green to detect *P. cinnamomi*, the pathogen of jarrah dieback disease, based on sequence variations at the *mtDNA* gene *Cox 2*.

As shown in Table 2, PCR-based methods (with or without additional steps) have been used as the main molecular approach for detecting oomycete pathogens infecting fiber crops. This pattern is similar to the detections of oomycete pathogens in other crops in general. A number of primers targeting different gene fragments have been explored as potential targets for PCR-based detections. Table 3 summarizes the genes and their primers that have been used for the detection and diagnostics of oomycete pathogens on fiber crops.

**Figure 2:**
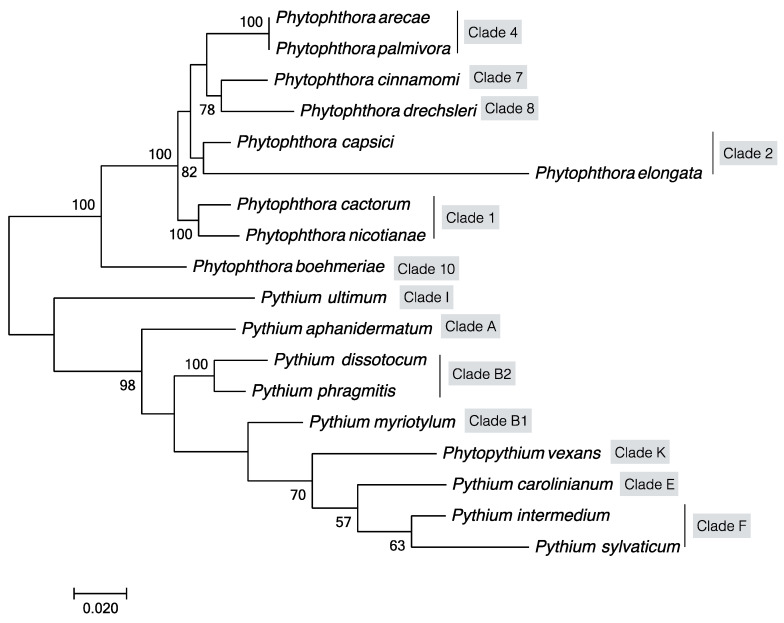
Maximum likelihood tree showing phylogenetic relationships among oomycete pathogens infecting fiber crops. The tree was constructed based on concatenated sequences of four gene fragments (LSU ribosomal RNA region, mitochondrial COI, SSU ribosomal RNA region and internal transcribed spacer (ITS) region) of oomycetes listed in Table 2. Statistical support for the branches was assessed by bootstrap with 1,000 replicates. Bootstrap values ≥50 are shown near the branch nodes.

With the advent of PCR amplification and the availability of DNA sequences of the above genes in oomycetes, there have been a number of phylogenetic studies of oomycetes [66,67,80]. For example, Briard et al. focused on *Pythium* and *Phytophthora* species using the ribosomal LSU sequences and showed that *P. vexans* was different from *Pythium* and *Phytophthora* [81]. Matsumoto et al. used ITS sequences and showed that species with filamentous and globose sporangia were phylogenetically separated [66]. Based on *cox2* gene sequences, Martin [80] showed that 60 isolates of *Pythium* belonging to 24 species formed three phylogenetic groups. Here, based on DNA sequences at four genes *LSU*, *COI*, *SSU* and ITS, we constructed a phylogeny among the oomycete pathogens known to infect fiber crops as listed in Table 2. Our analysis indicated that all fiber crop oomycete pathogens are clustered into two large clades corresponding to *Phytophthora* spp. and *Pythium* spp. (Figure 2), among which *P. palmivora* and *P. arecae*, *P. cactorum* and *P. nicotianae*, *P. dissotocum* and *P. phragmitis* were clustered in one clade with bootstrap values of 100%, respectively, consistent with previous studies [68]. Within each genus, we found several distinct clades, similar to those reported previously by Cooke et al. [82] and Lévesque et al. [83]. Specifically, our oomycete clades infecting fiber crops correspond to clades 1, 2, 4, 7, 8, and 10 of the genus *Phytophthora* and to clades A, B, E, F, K, and I of the genus *Pythium*. These results indicate that oomycete pathogens of fiber crops are evolutionary diverse. 

## 6. Molecular Identification of Oomycete Pathogens in Other Crops

As described above, a common molecular method for plant oomycete pathogen detection has been to used universal primers (e.g., ITS1 and ITS4 of the ITS regions) to first amplify the gene fragment, followed by sequencing and analyses of the amplified fragment. However, this process can be time-consuming and laborious. Therefore, developing species-specific primers targeting either the ITS or other regions that allow plant pathologists to directly detect certain pathogens would be more desirable. Indeed, the PvF1/PvR1 primer pair based on sequence variations within the ITS regions allowed fast and specific detection of *P. vexans* [85]. However, previous reports have indicated that the ITS regions have low sequence variability among many closely related *Phytophthora* species [86]. Consequently, species-specific primers are difficult to develop for many of the species in this genus. As a result, variable regions in several other genes have been used to design specific primers for the detection of such pathogens, including the putative storage protein gene *LPV* [87], *SSU rRNA* [88], GTP-binding protein (*Ypt1*) and the mitochondrial genes such as *Cox 1* and *Cox 2* [89]. In addition, Yuan et al. [90] showed that several mitochondrial genes *rpl6*, *rps10*, *atp8*, *nad11*, *rps11*, *rps2*, *rps3*, *nad9*, and *rps4* had similar sequence variations as the nuclear rDNA genes and that those mitochondrial genes could be used for the identification of pathogenic water mold species in the oomycete Class Peronosporales. Indeed, due to the high copy number and haploid nature of mitochondrial genomes in oomycetes (instead of the diploid nature of their nuclear genomes), there are increasing efforts to develop species-specific mitochondrial markers for detecting oomycetes.

As shown in Table 2 and Table 3, the most commonly used detection method for oomycetes infecting fiber crops is conventional PCR technology. Although conventional PCR has been useful for detecting *Phytophthora* and *Pythium* species in cultures, it has not been as successful when the pathogen count is low in diseased tissue samples. Early stages of infections and/or latent infections often contain low concentrations of pathogens and conventional PCR could miss the detections. Consequently, it is desirable to use more sensitive technologies such as LAMP, RT-qPCR, nested PCR, etc., that can detect oomycete pathogens in tissues in a timely manner, even when they are in low abundance (Table 4). Indeed, the ITS-based LAMP assay was found to be highly sensitive and specific for testing artificially and naturally infected plants by oomycete pathogens *P. capsici*, *P. ultimum* [91,92], with the limit of detection for *P. ultimum* at approximately 1 pg/μL DNA, which is 1000 times more sensitive than conventional PCR [92]. An increasing number of DNA fragments such as the mitochondrial *Cox 1* and *Cox 2* genes, and the nuclear *β-tubulin*, elicitin *ParA1* and the *Ypt1* genes have been explored as molecular markers for oomycete detections [30]. Together, by expanding the gene regions and the types of techniques, more sensitive and specific methods will be continuously developed that should allow fast and accurate detections of many oomycete pathogens, not only for those infecting fiber crops but also for other crops.

## 7. Conclusions and Future Prospects

As shown above, the main genera of oomycete pathogens infecting fiber crops are *Phytophthora* and *Pythium* (and *Phytopythium).* These two genera contain many species, with most species capable of infecting multiple species of host plants. Oomycete species in these two genera can produce abundant sporangia and zoospores to infect host plants and to spread among ecological niches. In addition, most of these pathogens can cause complex co-infections with other pathogens [40,41]. Due to their high similarity in morphology, many closely related species in these two genera are difficult to differentiate based on morphological and culture features. Additional challenges for identifying oomycete pathogens include: (i) symptoms of oomycete diseases are often very similar to those caused by other pathogens or non-biological reasons, and (ii) the slow growth of oomycete pathogens compared to many other microorganisms and pathogens. For example, diseases caused by *Pythium* spp. with symptoms such as stunting, yellowing, and rotting of plants in different parts, are very similar to the symptoms caused by nutrient deficiency and other root rot pathogens. In addition, in the case of complex infections by multiple organisms, although oomycetes may be the main pathogens, other fast-growing pathogens or contaminants may appear on culture media first and suppress the growth of oomycetes, resulting in incorrect diagnosis of disease agents. Thus, having sensitive and specific molecular tests are essential for early detection and diagnosis. In this review, we summarized the molecular markers that have been used to identify oomycete pathogens infecting fiber crops. Our review identified that several markers targeting fragments of genes such as *ITS*, *EF-1α*, *Cox 1* and *Cox 2*, can effectively help identify many species in both oomycete genera. However, most published detection technologies of oomycete on fiber crops rely on conventional PCR technology using universal PCR primers followed by sequencing of the amplified DNA fragments. Such a protocol often takes time and require significant starting materials that might not be present during early stages of infections and/or in the case of latent infection. Developing more efficient technologies such as LAMP assays that can be applied in field settings should significantly enhance the value of molecular diagnosis in the prevention and treatment of plant diseases caused by oomycete pathogens. Indeed, LAMP technology has been used to detect several oomycete pathogens (e.g., *P. capsici*, *P. ultimum*) infecting other crops, but not yet on fiber crops [91,92,97]. Few modifications are needed to adopt the existing LAMP technology from other crops to fiber crops [98].

Real-time monitoring of disease agents in crop fields provides essential information about the epidemiology of infectious diseases. Such epidemiological information can help farmers and agronomists develop effective control and prevention strategies against plant infectious diseases at local, regional, and national levels [99]. For example, at a local level, when farmers observe a possible disease in their crop fields, it would be highly beneficial for them to understand as soon as possible the underlying disease agent(s), the method by which the pathogen is spread, and the pesticides that these pathogens may be susceptible to, so that they can determine the best mitigation strategies. Obtaining such epidemiological information requires fast, sensitive, accurate, and cost-effective methods to monitor the pathogen in crop fields. Recently, researchers have developed a new technique that uses microneedle patches to collect oomycete DNA from plant tissues within one minute, rather than the hours needed based on conventional methods [100]. Additionally, the same team also developed a Smartphone-based sensor for volatile compounds for early detection of tomato late blight caused by *P. infestans* at two days after inoculation [101]. This approach allowed them to differentiate *P. infestans* from other pathogens that caused similar symptoms on tomato foliage [101]. In addition, the ability to predict disease outbreaks is an important goal in disease surveillance and pathogen detection. In this regard, NGS technology can provide abundant information and offer significant potential. For example, the NGS technology can be used to directly identify the samples without culture, including those that cannot be cultured and cannot be identified by other technologies [102]. Indeed, with decreasing cost and increasing accessibility of analytical platforms, NGS and genome-based species identification and detection could revolutionize the diagnosis of oomycete pathogens [2]. The development and application of these and other technologies will provide more efficient detection of oomycete pathogens in fiber crop fields. Together, we believe the future is bright for the efficient detection of oomycete pathogens in fiber crops.

## Figures and Tables

**Table 1:** Major types of commercial fiber crops and their distributions around the world.

Group	Crop	Main Distribution	Growth Habitat	Main Applications	Main Oomycete Diseases
Seed fiber	Cotton (*Gossypium hirsutum*)	China, USA, India, Brazil, Mexico	Thermophilic plant, sandy loam, loam and light clay with better heat transfer and permeability	Textiles, cottonseed oil	Cotton blight
	Sponge gourd (*Luffa cylindrica*)	China, Japan, Korea, India (Kerala, Andhra Pradesh)	Requires 150 to 200 warm days to mature	Used as a bath or kitchen sponge and food	Phytophthora fruit rot
Bast fiber	Hemp *(Cannabis sativa)*	China, Canada, USA, Europe, East Asia, Nepal	Grows at 16–27 °C, sufficient rain at the first six weeks of growth, short day length.	Textiles, hempseed oil, prescription drug	Hemp blight, hemp root and crown rot wilt
	Ramie *(Boehmeria nivea*)	China, Brazil, Philippines, India, Vietnam, Laos, Cambodia	Sandy soil and warm, wet climates, rainfall averaging at least 75 to 130 mm per month	Textiles, soil and water conservation, medicine	Ramie blight, ramie brown root rot
	Flax (*Linum usitatissimum*)	France, Russia, Netherlands, Belarus, Belgium, Canada, Kazakhstan, China, India	Well-drained loam and cool, moist temperate climates	Linen, flax yarn, flax seed, linseed oil	Flax root rot
Leaf fiber	Sisal (*Agava sisalana*)	Brazil, Tanzania, Kenya, Madagascar, China, Mexico, Haiti, Venezuela, Morocco, South Africa	In the tropical and temperate zones with mean temperature at 25 °C with sufficient sunshine	Making rope, twine, paper, cloth, wall covering and dartboards	Sisal zebra leaf disease
Grass fiber	Silvergrass (*Miscanthus sinensis*)	China, Japan, Korea, USA	In temperate regions around the world	Ornamental plant, bioenergy production	Basal stem rot and foliar blight
	Reed (*Phragmites australis*)	Northern Hemisphere	In lakes and rivershores, marshes, coastal brackish swamps, and lagoons	Used in phytoremediation, protecting shoreline from bank erosion, and serving as a food source or habitat protection for arthropods, birds and mammals.	Dieback of reed stands
Palm fiber	Windmill Palm (*Trachycarpus fortunei*)	China, Japan, India, Burma	Warm and humid climate	Making rope, coir raincoat, brown bandage, carpet, brush and filling material for sofa, medicine, ornament	Windmill Palm bud and root rot
Woody fiber	Jarrah (*Eucalyptus marginata*)	Australia	Rainfall isohyet exceeds 600 mm, grows in soils derived from ironstone	Structural material for bridges, wharves, railway sleepers, ship building and telegraph poles, medicine	Jarrah dieback

**Table 2:** Diseases of oomycetes on main fiber crops and identification methods.

Pathogens	Disease	Method	Marker	Host Plant	Geographic Region(s)	Reference
***Phytophthora* spp.**						
*P. arecae*	Sisal zebra spot disease	Conventional PCR	*ITS*	*Agava sisalana*	China, India	[34]
*P. boehmeriae* (Dominant pathogen)	Cotton blight	Conventional PCR	*ITS*	*Gossypium hirsutum*	China	[31]
*P. boehmeriae*	Ramie blight	Conventional PCR	*Cox 2, Nad 9*, *Rps 10, Sec Y*	*Boehmeria nivea*	China (Taiwan), Australia, Greece, South Africa	[32]
*P. cactorum*	Ramie blight	Morphological		*Boehmeria nivea*	Jiangxi, China	[35,36]
*P. cactorum*	Cotton blight	Conventional PCR	*ITS*	*Gossypium hirsutum*	China	[31]
*P. capsici*	Sponge gourd rot	Conventional PCR	*Cox 1, Cox 2, Nad 1*, *Nad 5, 𝛽-tubulin*, *EF1, Enolase, HSP090, Ura3, ITS*	*Luffa cylindrica*	USA	[37]
*P. cinnamomi*	Jarrah dieback	Quantitative real-time PCR	*Cox 2*	*Eucalyptus marginata*	Western AustraliaUSA	[38,39,40]
*P. drechsleri*	Cotton blight	Conventional PCR	*ITS*	*Gossypium hirsutum*	China	[31]
*P. elongata*	Jarrah dieback	Conventional PCR	*ITS and* *Cox 1*	*Eucalyptus marginata*	Western Australia	[41]
*P. nicotianae*	Cotton blight	Conventional PCR	*ITS*	*Gossypium hirsutum*	China	[31]
*P. nicotianae*	Windmill palm bud and root rot	Conventional PCR	*ITS*	*Trachycarpus fortunei*	eastern Sicily, Italy	[42]
*P. nicotianae* (Dominant pathogen)	Sisal zebra spot disease	Conventional PCR	*ITS*	*Agava sisalana*	China, India	[34]
*P. palmivora*	Cotton blight	Conventional PCR	*ITS*	*Gossypium hirsutum*	China	[31]
*P. palmivora*	Sisal zebra spot disease	Conventional PCR	*ITS*	*Agava sisalana*	China, India	[34]
*P. palmivora*	Windmill palm bud and root rot	Conventional PCR	*ITS*	*Trachycarpus fortunei*	eastern Sicily, Italy	[42]
***Pythium* spp.**						
*P. aphanidermatum*	Bush okra damping-off	Morphological		*Corchorus olitorius*	Egypt	[43]
*P. aphanidermatum*	Hemp root rot and crown wilt	Conventional PCR	*ITS*	*Cannabis sativa*	California, USA	[44]
*P. aphanidermatum*	Hemp crown and root Rot	Conventional PCR	*ITS*	*Cannabis sativa*	Indiana, USA	[45]
*P. baryanum*	Cotton damping-off	Morphological		*Gossypium hirsutum*	Egypt	[46]
*P. carolinianum*	Cotton root rot	Morphological		*Gossypium hirsutum*	Egypt	[47]
*P. dissotocum*	Marijuana root rot	Conventional PCR	*ITS, EF-1α*	*Cannabis sativa*	Canada	[48]
*P. intermedium*	Flax root rot	Taxonomic		*Linum usitatissimum*	UK	[49]
*P. myriotylum*	Marijuana root rot	Conventional PCR	*ITS, EF-1α*	*Cannabis sativa*	Canada	[48]
*P. myriotylum*	Hemp root rot and Wilt	Conventional PCR	*ITS, Cox 1, Cox 2*	*Cannabis sativa*	Connecticut, USA	[50]
*P. phragmitis*	Reed die-back syndrome	Conventional PCR	*ITS and* *Cox 2*	*Phragmites australis*	Lake Constance, Germany	[51]
*P. sylvaticum*	Silvergrass stem rot and blight	Conventional PCR	*ITS, Cox 2*	*Miscanthus sinensis*	Illinois, USA	[52]
*P. ultimum*	Flax damping off	Morphological		*Linum usitatissimum*	India	[53]
*P. ultimum*	Hemp crown and root rot	Conventional PCR	*ITS*	*Cannabis sativa*	Indiana, USA	[54]
*P. ultimum*	Cotton damping-off	Morphological		*Gossypium hirsutum* L.	Egypt	[55]
*P. vexans*	Ramie brown root rot	Conventional PCR	*ITS, 18S, 28S*	*Boehmeria nivea*	China	[33]
*Pseudoperonospora cannabinus*	Hemp mildew	Morphological		*Cannabis sativa*	Austria, Canada, China, Italy	[56]

**Table 3:** Genes and PCR primers used for their amplification of oomycete infecting fiber crops.

Target DNA	Primer Name and Sequence (5′-3′)		Pathogens	TM (°C)	Product (bp)	Reference
***ITS1-5.8S-ITS2***	DC6	GAGGGACTTTTGGGTAATCA	*Phytophthora* spp., *Pythium* spp.	62	1300	[31,84]
	ITS4	TCCTCCGCTTATTGATATGC				
	PB1	CGGCTTTCGGGCTGCTGC	*P. boehmeriae*	62	750	[31]
	PB2	ATACCCGAAGGCAAAGCGC				
	ITS1-F	CTTGGTCATTTAGAGGAAGTAA	*P. aphanidermatum*	60	700	[44,48]
	ITS4	TCCTCCGCTTATTGATATGC	*P. dissotocum*, *P. myriotylum*			
	ITS6	GAAGGTGAAGTCTAACAAGG	*P. cinnamomi*, *P. palmivora*, *P. elongate*	55	796–910	[42,51]
	ITS4	TCCTCCGCTTATTGATATGC				
	ITS1	TCCGTAGGTGAACCTGCGG	*P. vexans*,	55	810–900	[33,42]
	ITS4	TCCTCCGCTTATTGATATGC	*P. nicotianae*			
*rDNA 18S*	NS3	GCAAGTCTGGTGCCAGCAGCC	*P. vexans*	50–52	610	[33]
	NS4	CTTCCGTCAATTCCTTTAAG				
*rDNA 28S*	LR0R	GTACCCGCTGAACTTAAGC	*P. vexans*	50–52	810	[33]
	LR3	CCGTGTTTCAAGACGGG				
*Cox 1*	FM82	TTGGCAATTAGGTTTTCAAGATCC	*P. elongate*	56	742	[41]
	FM83	CTCCAATAAAAAATAACCAAAAATG				
*Cox 2 (RT-qPCR)*	PCIN147F	CCAGCAACTGTTGTGCATGG	*P. cinnamomi*	55–60	100	[38]
	PCIN249R	AATATAATAAAGCAAATGATGGT				
	PCIN146F	TCCAGCAACTGTTGTGCATG				
	PCIN250R	GAATATAATAAAGCAAATGATGGT				
	PCIN147F	CCAGCAACTGTTGTGCATGG				
	PCIN246R	ATAATAAAGCAAATGATGGT				
	PCIN150F	GCAACTGTTGTGCATGGAGC				
	PCIN247R	TATAATAAAGCAAATGATGGT				
*Cox 2*	FM35	CAGAACCTTGGCAATTAGG	*P. phragmitis*	-	563	[51]
	FM58	CCACAAATTTCACTACATTG				
	FM58	CCACAAATTTCACTACATTG	*P. sylvaticum*	56	544	[52]
	FM66	TAGGATTTCAAGATCCTG				
*EF-1α*	EF-1	ATG GGT AAG GAGGAC AAG AC	*P. dissotocum*,	60	700	[48]
	EF-2	GGA GGT ACC AGTGAT CAT GTT	*P. myriotylum*			

**Table 4:** Main techniques used for detecting oomycetes.

Pathogens	Method	Marker		Primer (5′-3′)	Sample	Hosts	Reference
*P. capsici*	RT-qPCR	*Actin*	YM2F	ATTCCTCCTGATAGATAG	Mycelia		[93]
			YM2R	CCCTCATCACAGAATGC			
*P. capsici*	Nested PCR	*Ypt1*	Ypt1F	ACGGAGAGCTACATCTCGAC	Mycelia		[88]
			Ypt1R	GTCAGATCGCTCTTGTTACC			
			PcYpt1F	AGACTCTGTTGTATAGCAGAG			
			PcYpt1R	AACGTCTTGAACTTTGGTTG			
*P. capsici*	LAMP	*ITS*	F3	GCTGCGGCGTTTAAAGGA	Leaves	Pepper	[91]
			B3	AGTGCACACAAAGTTCCCAA			
			FIP	ACGCCACAGCAGGAAAAGCATTGAGTGTTCGATTCGCGGTA			
			BIP	GGCTTGGCTTTTGAATCGGCTTTGGATCGACCCTCGACAG			
*P. cinnamomi*	SYBR green (nested PCR)	*LPV*	LPV3-fwd	GTGCAGACTGTCGATGTG		Avocado	[86]
			LPV3-rev	GAACCACAACAGGCACGT			
			LPV3N-fwd	GTCACGACCATGTTGTTG			
			LPV3N-rev	GAGGTGAAGGCTGTTGAG			
*P. nicotianae*	Duplex-PCR	*SCAR*	MPhnic 2F	TTCGAGAAGTACGTGGCGTTT	Leaves	kalanchoe	[94]
			MPhnic 2R	TTGCAGCGGAGAGTGAGAACT			
			MPhnic 3F	ATCTCCCAATCGACCGTGAA			
			MPhnic 3R	CAAGCACGTGACTCGGTTGA			
			MPhnic 5F	CTCGATACGGACGCAAAGGT			
			MPhnic 5R	CATGGCTACAGCTGCTGCAA			
*P. ultimum*	Conventional PCR	*ITS*	PuF	ATGATGGACTAGCTGATGAA	Soils	American ginseng	[95]
			PuR	TTCCATTACACTTCATAGAA			
			Pu1F1	GACGAAGGTTGGTCTGTTG	Tubers	Potato	[96]
			Pu2R1	CAGAAAAAGAAAGGCAAGTTTG			
*P. ultimum*	TaqMan	*ITS*	92F	TGTTTTCATTTTTGGACACTGGA	Tubers	Potato	[96]
			166R	TCCATCATAACTTGCATTACAACAGA			
			116T	FAM-CGGGAGTCAGCAGGACGAAGGTTG-VIC			
*P. ultimum*	LAMP	*ITS*	F3	CAACTGGAAAAGCAAGCGG	Leaf	Wheat, soybean, cucumber, and tobacco	[92]
			B3	CCGAAGAACTGTGTCCGC			
			FIP	GAGCCAGACGGGCCAGTATCAAGTTACAGTGGCGTTGTCA			
			BIP	TCTCTGTTGCTCGACTGGAGGGTTCCACCTCCTGTAAGACCT			
			F-Loop	GCTTGCTCCAGTACGAATGC			
*P. vexans*	TaqMan	*ITS*	PvF1	TTTCCGTTTTGTGCTTGATG			[84]
			PvR1	AGCGAACACACCCAATAAGC			
			VexP1	HEX™-CCGTGTCTGCTGGCGGGTC-Iowa Black^®^ FQ			
*P. vexans*	RT-qPCR	*SSU*	VexansF2	TATACAACCTTGATCGAC	Root tissue	Peach	[87]
			VexansR2	GATGGAAAATTGCAACC			
